# A Population-Based Cohort Study on the Association Between Oral Third-Generation Cephalosporins and Other Antimicrobial Prescriptions and Adverse Events: Findings From the Shizuoka Kokuho Database Study

**DOI:** 10.7759/cureus.78923

**Published:** 2025-02-12

**Authors:** Hisashi Dote, Daito Funaki, Yoshikazu Ichikawa, Nanako Ubukata, Hiromu Miyake, Akinori Miyakoshi, Michiko Oshima, Emi Ohata, Yutaro Imaichi, Aya Shoji-Asahina, Eiji Nakatani

**Affiliations:** 1 Department of Emergency and Critical Care Medicine, Seirei Hamamatsu General Hospital, Hamamatsu, JPN; 2 Graduate School of Public Health, Shizuoka Graduate University of Public Health, Shizuoka, JPN; 3 Department of Biostatistics and Health Data Science, Graduate School of Medical Sciences, Nagoya City University, Nagoya, JPN

**Keywords:** antibiotics, clostridioides difficile infections, drug resistance, epidemiology, receipt database

## Abstract

Introduction: The rise of drug-resistant bacteria and associated adverse events have been linked to inappropriate antibiotic use. In Japan, inappropriate prescriptions of oral antimicrobials might be prevalent and contribute to this issue. This study explored the association between oral third-generation cephalosporins and other antimicrobials with adverse events in the Japanese population.

Methods: We conducted a population-based cohort study using a large-scale database, i.e., the Shizuoka Kokuho Database. This study included individuals with health checkup records, with the observation period for each participant ranging from insurance enrollment or April 2012 to insurance withdrawal or September 2020. The primary outcome was hospitalization with clinically important antibiotic-related adverse events (ciArAEs) based on the International Classification of Diseases, Tenth Revision (ICD-10) codes. In addition, we executed a multivariable analysis employing potential predictive factors selected from comorbidities, prescribed antimicrobials, and health checkup results.

Results: Of the 685,161 individuals included in the analysis, 2,557 had ciArAEs. Third-generation cephalosporins (HR: 1.14, 95% CI: 1.01-1.29), tetracyclines (HR: 2.14, 95% CI: 1.47-3.13), and aminoglycosides (HR: 8.36, 95% CI: 1.18-59.2) were identified as potential predictive factors for ciArAEs among oral antimicrobial agents. Additional predictive factors included older age, males, intravenous penicillin, and various comorbidities.

Conclusions: By utilizing a large-scale database, we demonstrated the relationship between the use of antimicrobial agents, including oral third-generation cephalosporins, and ciArAEs. This finding underscores the need for enhanced prescription practices and further antimicrobial resistance and adverse events studies.

## Introduction

Recently, the rise in drug-resistant bacteria and the subsequent expansion of healthcare-associated infections have become urgent global issues in healthcare [1]. As a countermeasure against drug-resistant microbes, antimicrobial stewardship, including promoting the proper use of antimicrobial agents, occupies an important position [2,3]. In Japan, it is estimated that oral antibiotics constitute more than 90% of all antibiotic prescriptions [4]. Reports from Japan indicate that many third-generation cephalosporins, macrolides, and quinolones are prescribed for common infections such as respiratory and gastrointestinal infections, which are generally considered inappropriate [5]. Accordingly, it is essential to address the proper use of oral antibiotics in Japan.

Inadequate prescription of oral antibiotics is associated with an increase in drug-resistant bacterial infections [6-8]. When antibiotics are inappropriately prescribed, the therapeutic effect cannot be expected. In addition to drug-resistant infections, other adverse events such as organ disorders and *Clostridioides difficile* infections (CDIs) may occur in patients due to this practice [9,10]. However, the patterns of use of oral antibiotics and the occurrence of adverse events related to oral antibiotics in Japan have not been fully understood.

Among these, oral third-generation cephalosporins are often cited as classic examples of inappropriate antimicrobial use, primarily due to their low bioavailability and unnecessarily broad antibacterial spectrum [11]. Therefore, we employed a large, community-based database to examine prescribing patterns and to confirm whether clinically important antibiotic-related adverse events (ciArAEs) occur more frequently in patients with a history of such prescriptions.

This study aims to investigate the prescribing patterns of oral antibiotics in Japan, assess the association between prescriptions for oral third-generation cephalosporins and other antimicrobials with ciArAEs, and apply the findings to inform policies for optimizing antibiotic use and mitigating antimicrobial resistance. These insights are crucial for guiding future strategies, emphasizing the appropriate use of antibiotics, and ultimately mitigating the emergence of drug-resistant bacteria.

## Materials and methods

Database

This study utilized the Shizuoka Kokuho Database (SKDB). The SKDB includes subscribers of the National Health Insurance and Later Stage Elderly Medical Care System in Shizuoka prefecture. Over 8.5 years from April 2012 to September 2020, 2,654,567 patients were included. The SKDB contains information such as disease names, medical receipts, nursing care benefits, and results of specific health examinations. In addition to basic information like age and sex, information such as disease names by the International Classification of Diseases, Tenth Revision (ICD-10) codes, history of prescription drugs, medical examinations, and results of inquiries can be obtained [12].

Japanese medical insurance and health checkup systems

Japan's medical system is based on a comprehensive insurance structure. In Japan, two types of health insurance are provided for people aged <75 years: Employee Health Insurance for employees of large companies and government organizations, and National Health Insurance for small business owners and their employees. Additionally, health insurance for people aged ≥75 years is provided by the Latter Stage Elderly Medical Care System.

The Japanese Ministry of Health, Labour, and Welfare recommends annual health checkups for insured individuals 40 years of age and older, with a particular emphasis on visceral fat obesity. All health checkup data required for the study, including age, sex, height, weight, systolic and diastolic blood pressure, smoking habits, and the use of antihypertensive, lipid-lowering, and hypoglycemic drugs, were available through self-reported questionnaire data. In Shizuoka Prefecture, the participation rate for these medical checkups was 52.9% in 2015.

Study design, data available period, and study population

**Figure 1:**
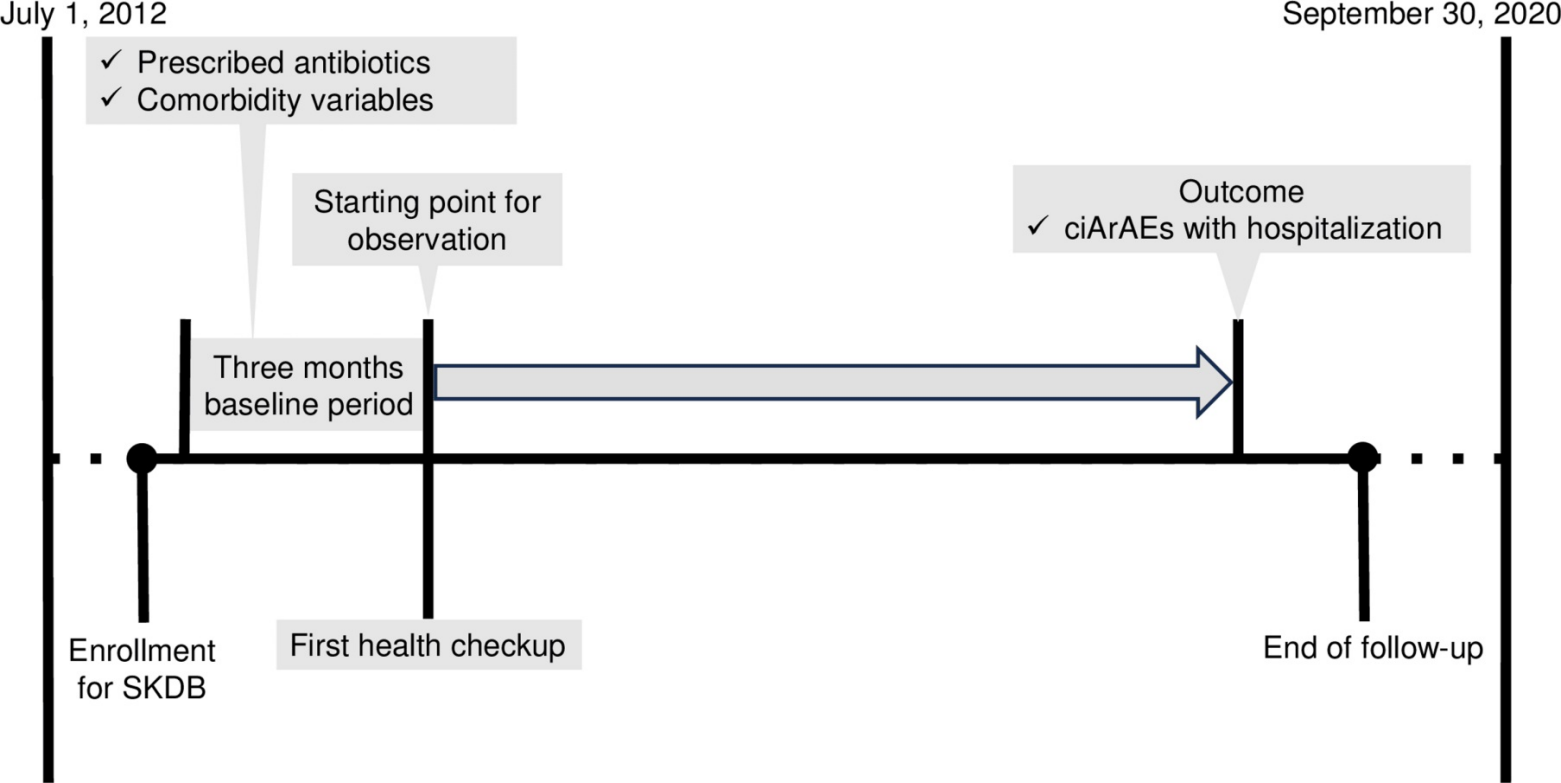
Study schema. The questionnaire mentioned in Figure 1 was part of a routine medical checkup and was not specifically tailored or designed for the purposes of the present research study. SKDB: Shizuoka Kokuho Database; ciArAEs: clinically important antibiotic-related adverse events.

The study scheme is shown in Figure 1. The study was analyzed as a retrospective cohort generated from the database. The dataset comprised 8.5 years of longitudinal data from April 2012 to September 2020. All enrollees were investigated using individually linked data in the databases for their annual health checkups and insurance claims. Each enrollee's data availability period was defined as the date of insurance registration or April 2012, whichever was later, to the date of insurance withdrawal or September 2020, whichever was earlier. We extracted the records of the first health checkup during the observation period. The baseline period was defined as the three months preceding the date of the first health checkup. Antimicrobials prescribed during the baseline period were used as variables. Participants with an observation period less than three months before the date of health checkups were excluded.

Hospitalization with antibiotic-related adverse events

Patients were classified as hospitalized with ciArAEs when the relevant codes in Appendix 1 from the ICD-10 appeared in their insurance claims. This definition encompasses not only cases where ciArAEs are the primary reason for hospitalization but also those that arise during the hospital stay. Whereas we acknowledge that not all hospitalizations are primarily owing to ciArAEs, our goal was to document each occurrence during hospital stays, ensuring a comprehensive understanding of antibiotic-related adverse events in the inpatient setting.

In our study, we have meticulously defined ciArAEs to exclusively cover infections caused by drug-resistant bacteria and CDIs, recognizing their grave implications on both clinical settings and public health. While we acknowledge the spectrum of adverse events associated with antimicrobials, ranging from skin eruptions and allergic reactions to more severe outcomes, we have deliberately chosen to concentrate on these specific severe outcomes. This strategic focus is grounded in our commitment to tackle the urgent clinical challenges associated with antimicrobial prescriptions, aiming to generate robust evidence that will guide the reduction of inappropriate oral antimicrobial use. By narrowing down our attention to these severe adverse events, which are frequently discussed yet under-represented in empirical clinical research, we aim to fill a critical gap in the current understanding and management of antimicrobial-related risks.

On the basis of a previous study showing that the association between antimicrobial prescriptions and subsequent antibiotic resistance persists for up to one year [13], we conservatively chose a longer follow-up period in this study.

Covariates

Comorbidity variables, based on the Charlson Comorbidities [14], were extracted using the ICD-10 codes in Appendix 2. Prescriptions for antibiotics were extracted using drug codes based on therapeutic category codes. The antibiotics were classified according to the categories commonly used clinically, and the routes of administration (oral and intravenous) were also distinguished. Furthermore, age, sex, and body mass index (BMI) [15] were extracted from the results of the health checkups.

Statistical analysis

Data were summarized as means and standard deviations for continuous variables, and frequencies and percentages for categorical variables. To compare the two groups, the chi-squared test was used for categorical analysis. Univariable and multivariable Cox proportional hazards regression were performed to explore factors associated with outcomes. Hazard ratios (HRs), 95% confidence intervals (CIs), and p-values were calculated. We used Spearman’s correlation coefficient to assess multicollinearity, and when two variables had an absolute correlation coefficient >0.4, we included only one in the final model based on clinical relevance. Simple imputation of missing data was not performed, as missing data do not arise completely randomly from all participants. A p-value of < 0.05 was considered statistically significant. All analyses were performed using the software package SAS version 9.4 (SAS Institute Inc., Cary, NC, USA).

Ethics

To protect participant confidentiality, all enrollee data were anonymized by the Federation of National Health Insurance Association. This study adhered to the principles of the Declaration of Helsinki and was approved by the Medical Ethics Committee of Shizuoka Graduate School of Public Health, Shizuoka, Japan (# SGUPH_2021_001_048).

## Results

Study population and baseline characteristics

**Figure 2:**
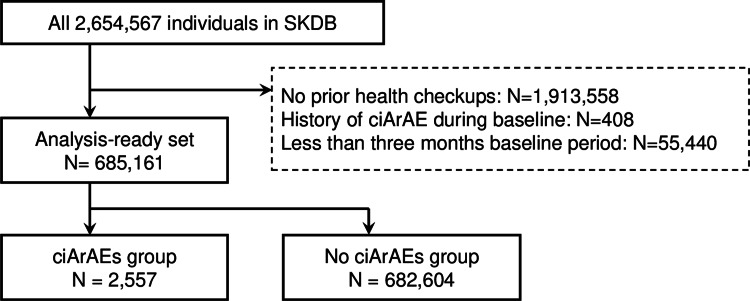
Flow diagram of our study. SKDB: Shizuoka Kokuho Database; ciArAEs: clinically important antibiotic-related adverse events.

Among the 2,654,567 individuals enrolled in SKDB, those who lacked a three-month baseline period, had no health examination records, or had a prior history of ciArAE during the baseline were excluded. Consequently, 685,161 individuals were included in the final analysis (Figure 2). The median (maximum) duration of follow-up was 6.21 (8.25) years, and 2,557 participants (0.37%) were hospitalized due to ciArAE during the observation period.

Participants were stratified into two groups based on the presence or absence of ciArAE-related hospitalizations, with details provided in Table 1. The ciArAE group had a higher proportion of older adults and men, whereas BMI was comparable between the groups. Furthermore, every comorbidity assessed was more frequently observed in the ciArAE group.

**Table 1:** Demographics of participants by ciArAEs status. Categorical variables are summarized as frequency (percentage). ciArAEs: clinically important antibiotic-related adverse events; IV: intravenous injection; MRSA: methicillin‐resistant *Staphylococcus aureus*.

Variable	Category or unit	ciArAEs group	No ciArAEs group
		(n = 2,557)	(n = 682,604)
Age	0 to <40 years	2 (0.1)	5,749 (0.8)
	40 to <50 years	25 (1.0)	52,964 (7.8)
	50 to <60 years	71 (2.8)	64,680 (9.5)
	60 to <70 years	529 (20.7)	257,842 (37.8)
	70 to <80 years	1,023 (40.0)	205,803 (30.1)
	80 to <90 years	812 (31.8)	85,747 (12.6)
	≥90 years	95 (3.7)	9,819 (1.4)
Sex	Male	1,376 (53.8)	292,994 (42.9)
Body mass index	<18.5	267 (10.5)	62,408 (9.2)
	18.5 to <25.0	1,699 (67.0)	466,100 (68.4)
	25.0 to <30.0	504 (19.9)	133,011 (19.5)
	≥30.0	64 (2.5)	19,441 (2.9)
	Missing number	23	1,644
Comorbidities extracted between three months preceding the first health checkup date			
Congestive heart failure	Presence	411 (16.1)	43,760 (6.4)
Myocardial infarction	Presence	51 (2.0)	8,011 (1.2)
Peripheral vascular disease	Presence	376 (14.7)	44,389 (6.5)
Cerebrovascular disease	Presence	558 (21.8)	72,723 (10.7)
Dementia	Presence	184 (7.2)	13,742 (2.0)
Chronic pulmonary disease	Presence	485 (19.0)	81140 (11.9)
Rheumatic disease	Presence	148 (5.8)	15,985 (2.3)
Peptic ulcer disease	Presence	520 (20.3)	75,381 (11.0)
Liver disease	Presence	397 (15.5)	70,696 (10.4)
Diabetes	Presence	189 (7.4)	24582 (3.6)
Hypertension	Presence	1,574 (61.6)	292,961 (42.9)
Hemiplegia or paraplegia	Presence	30 (1.2)	2,591 (0.4)
Renal disease	Presence	111 (4.3)	10,696 (1.6)
Any malignancy	Presence	285 (11.1)	38,306 (5.6)
Prescribed antimicrobials within three months preceding the first health checkup date			
Penicillins (oral)	Prescribed	27 (1.1)	8,518 (1.2)
Penicillins (IV)	Prescribed	15 (0.6)	1,185 (0.2)
Penicillins with beta-lactamase inhibitors (oral)	Prescribed	7 (0.3)	1,682 (0.2)
Penicillins with beta-lactamase inhibitors (IV)	Prescribed	4 (0.2)	430 (0.1)
First-generation cephalosporins (oral)	Prescribed	22 (0.9)	5,253 (0.8)
First-generation cephalosporins (IV)	Prescribed	16 (0.6)	2,501 (0.4)
Second-generation cephalosporins (oral)	Prescribed	6 (0.2)	1,304 (0.2)
Second-generation cephalosporins (IV)	Prescribed	16 (0.6)	3,326 (0.5)
Third-generation cephalosporins (oral)	Prescribed	306 (12.0)	63,980 (9.4)
Third-generation cephalosporins (IV)	Prescribed	31 (1.2)	3,375 (0.5)
Fourth-generation cephalosporins (IV)	Prescribed	1 (0.0)	311 (0.0)
Cephalosporin with beta-lactamase inhibitors (IV)	Prescribed	4 (0.2)	489 (0.1)
Carbapenems (oral)	Prescribed	13 (0.5)	1,685 (0.2)
Carbapenems (IV)	Prescribed	11 (0.4)	898 (0.1)
Aminoglycosides (oral)	Prescribed	1 (0.0)	31 (0.0)
Aminoglycosides (IV)	Prescribed	76 (3.0)	14,125 (2.1)
Quinolones (oral)	Prescribed	210 (8.2)	41,149 (6.0)
Quinolones (IV)	Prescribed	0 (0.0)	152 (0.0)
Anti-MRSA (oral)	Prescribed	0 (0.0)	3 (0.0)
Anti-MRSA (IV)	Prescribed	0 (0.0)	33 (0.0)
Macrolides (oral)	Prescribed	231 (9.0)	46,382 (6.8)
Macrolides (IV)	Prescribed	0 (0.0)	16 (0.0)
Lincomycin (oral)	Prescribed	1 (0.1)	135 (0.0)
Lincomycin (IV)	Prescribed	12 (0.5)	2,018 (0.3)
Tetracyclines (oral)	Prescribed	28 (1.1)	2,683 (0.4)
Tetracyclines (IV)	Prescribed	5 (0.2)	357 (0.1)
Glycylcycline (IV)	Prescribed	0 (0.0)	0 (0.0)
Nitroimidazoles (oral)	Prescribed	0 (0.0)	47 (0.0)
Sulfonamides (oral)	Prescribed	5 (0.2)	840 (0.1)
Sulfonamides (IV)	Prescribed	0 (0.0)	0 (0.0)
Monobactams (IV)	Prescribed	0 (0.0)	2 (0.0)
Antifungals (oral)	Prescribed	16 (0.6)	3174 (0.5)
Antifungals (IV)	Prescribed	0 (0.0)	13 (0.0)
Antituberculosis (oral)	Prescribed	6 (0.2)	512 (0.1)
Antituberculosis (IV)	Prescribed	0 (0.0)	13 (0.0)
Others (oral)	Prescribed	14 (0.5)	1,927 (0.3)
Others (IV)	Prescribed	17 (0.7)	1,778 (0.3)

Antibiotic prescriptions and incidence of ciArAEs

During the three-month baseline period, oral third-generation cephalosporins were prescribed to approximately 10% of the overall study population, followed by fluoroquinolones (8%), macrolides (6%), and penicillins (around 5%). Tetracyclines, sulfonamides, and aminoglycosides were prescribed less frequently (each under 3%). Intravenous (IV) antibiotics, especially penicillins and cephalosporins, were prescribed predominantly in hospital settings to about 1% of individuals. Overall, 2,557 participants experienced at least one ciArAE-related hospitalization. Although the majority of these ciArAE cases resulted from infections with antibiotic-resistant pathogens, CDI accounted for 23.4% (n = 598) of cases (Table 2).

**Table 2:** Number of each event of ciArAEs with hospitalization. ciArAEs: clinically important antibiotic-related adverse events; BLNAR: β-lactamase-negative ampicillin resistant; ESBL: extended-spectrum β-lactamase; MDR: multi-drug resistance; MRSA: methicillin‐resistant *Staphylococcus aureus*; PCG: penicillin-G; PRSP: penicillin-resistant *Streptococcus pneumoniae*; VCM: vancomycin; VRE: vancomycin-resistant enterococci.

Outcome	Number of cases (% of 2,557 events)
BLNAR infection	1 (0.04)
*Clostridium difficile* infection	599 (23.43)
ESBL	24 (0.94)
MDR bacterial infection	8 (0.31)
MRSA infection	1974 (77.2)
PCG resistant	2 (0.08)
PRSP infection	1 (0.04)
VCM resistant	4 (0.16)
VRE infection	4 (0.16)

Clinically important antibiotic-related adverse events (ciArAEs)

To identify potential predictors of ciArAEs, a univariate Cox proportional hazards regression analysis was performed (Table 3). Advanced age (≥65 years), male sex, and comorbid conditions such as congestive heart failure, diabetes, and renal disease demonstrated significant positive associations with ciArAEs. Regarding antimicrobial use, oral third-generation cephalosporins, tetracyclines, aminoglycosides, and intravenous penicillins were each significantly linked to an elevated risk.

**Table 3:** Univariate Cox regression analysis. IV: intravenous injection.

Variable	Category	Univariate Cox regression analysis			
		Hazard ratio	95% confidence intervals		p-value
Age	Aged 75 and over	3.60	3.32	3.89	<0.001
Sex	Male	1.67	1.54	1.80	<0.001
Body mass index	<18.5	1.23	1.08	1.40	0.002
	18.5 to <25.0	Reference			
	25.0 to <30.0	1.08	0.98	1.19	0.140
	≥30.0	1.03	0.80	1.32	0.800
Comorbidities					
Congestive heart failure	Presence	3.06	2.75	3.40	<0.001
Myocardial infarction	Presence	1.86	1.41	2.46	<0.001
Peripheral vascular disease	Presence	2.42	2.17	2.69	<0.001
Cerebrovascular disease	Presence	2.33	2.12	2.56	<0.001
Dementia	Presence	4.77	4.11	5.55	<0.001
Chronic pulmonary disease	Presence	1.78	1.62	1.97	<0.001
Rheumatic disease	Presence	2.57	2.18	3.03	<0.001
Peptic ulcer disease	Presence	1.96	1.78	2.16	<0.001
Liver disease	Presence	1.57	1.41	1.75	<0.001
Diabetes	Presence	2.25	1.94	2.61	<0.001
Hypertension	Presence	2.06	1.90	2.23	<0.001
Hemiplegia or paraplegia	Presence	3.24	2.26	4.64	<0.001
Renal disease	Presence	3.37	2.79	4.08	<0.001
Any malignancy	Presence	2.25	1.99	2.54	<0.001
Prescribed antimicrobials					
Penicillins (oral)	Prescribed	0.94	0.64	1.37	0.730
Penicillins (IV)	Prescribed	3.64	2.19	6.04	<0.001
Penicillins with beta-lactamase inhibitors (oral)	Prescribed	1.32	0.63	2.77	0.470
Penicillins with beta-lactamase inhibitors (IV)	Prescribed	2.80	1.05	7.46	0.040
First-generation cephalosporins (oral)	Prescribed	1.19	0.78	1.81	0.420
First-generation cephalosporins (IV)	Prescribed	1.62	0.99	2.65	0.050
Second-generation cephalosporins (oral)	Prescribed	1.22	0.55	2.72	0.630
Second-generation cephalosporins (IV)	Prescribed	1.26	0.77	2.07	0.350
Third-generation cephalosporins (oral)	Prescribed	1.30	1.15	1.46	<0.001
Third-generation cephalosporins (IV)	Prescribed	2.65	1.86	3.77	<0.001
Fourth-generation cephalosporins (IV)	Prescribed	0.93	0.13	6.59	0.940
Cephalosporin with beta-lactamase inhibitors (IV)	Prescribed	2.31	0.87	6.13	0.090
Carbapenems (oral)	Prescribed	2.03	1.17	3.49	0.010
Carbapenems (IV)	Prescribed	3.38	1.87	6.10	<0.001
Aminoglycosides (oral)	Prescribed	11.7	1.66	83.1	0.010
Aminoglycosides (IV)	Prescribed	1.36	1.08	1.71	0.010
Quinolones (oral)	Prescribed	1.36	1.18	1.56	<0.001
Macrolides (oral)	Prescribed	1.33	1.16	1.52	<0.001
Lincomycin (oral)	Prescribed	1.94	0.27	13.8	0.510
Lincomycin (IV)	Prescribed	1.47	0.83	2.59	0.180
Tetracyclines (oral)	Prescribed	2.81	1.93	4.07	<0.001
Tetracyclines (IV)	Prescribed	3.56	1.49	8.55	0.004
Sulfonamides (oral)	Prescribed	2.06	0.86	4.96	0.110
Antifungals (oral)	Prescribed	1.24	0.76	2.02	0.400
Antituberculosis (oral)	Prescribed	3.74	1.68	8.32	0.001
Others (oral)	Prescribed	1.96	1.16	3.32	0.010
Others (IV)	Prescribed	2.35	1.46	3.79	<0.001

Several variables showed correlation coefficients below 0.4 (Table 4). Variables with a p-value <0.05 in the univariate analysis were subsequently included in the multivariate model.

**Table 4:** Spearman's correlation coefficient. * Any malignancy, including lymphoma, leukemia, and malignant neoplasm of the skin. IV: intravenous injection; BLI: beta-lactamase inhibitors.

	Age	Sex	Congestive heart failure	Myocardial infarction	Peripheral vascular disease	Cerebrovascular disease	Dementia	Chronic pulmonary disease	Rheumatic disease	Peptic ulcer disease	Liver disease	Diabetes	Hypertension	Hemiplegia or paraplegia	Renal disease	Any malignancy*	Penicillins (IV)	Penicillin BLI (IV)	Third cephem (oral)	Third cephem (IV)	Carbapenem (oral)	Carbapenem (IV)	Aminoglycosides (oral)	Aminoglycosides (IV)	Quinolones(oral)	Macrolides(oral)	Tetracyclines (oral)	Tetracyclines (IV)	Other (oral)	Other (IV)
Age	1.00	-0.07	0.19	0.06	0.14	0.21	0.18	0.10	0.04	0.11	0.06	0.05	0.27	0.02	0.09	0.11	0.02	0.01	0.01	0.02	0.00	0.02	0.00	0.01	0.01	0.00	0.00	0.01	0.00	0.01
Sex	-0.07	1.00	-0.04	-0.07	-0.01	-0.04	0.02	-0.01	0.05	-0.03	-0.05	-0.05	-0.05	-0.02	-0.05	-0.08	-0.01	-0.01	0.01	-0.01	0.00	-0.01	0.00	0.01	0.03	0.02	0.00	0.00	0.01	0.01
Congestive heart failure	0.19	-0.04	1.00	0.20	0.13	0.15	0.08	0.10	0.04	0.12	0.07	0.09	0.22	0.02	0.14	0.06	0.03	0.02	0.01	0.02	0.00	0.01	0.00	0.00	0.02	0.01	0.00	0.01	0.00	0.01
Myocardial infarction	0.06	-0.07	0.20	1.00	0.08	0.06	0.01	0.02	0.01	0.07	0.02	0.06	0.10	0.01	0.05	0.02	0.01	0.00	0.01	0.01	0.00	0.01	0.00	0.00	0.00	0.00	0.00	0.00	0.00	0.00
Peripheral vascular disease	0.14	-0.01	0.13	0.08	1.00	0.15	0.03	0.07	0.06	0.10	0.07	0.09	0.15	0.02	0.06	0.04	0.01	0.01	0.02	0.01	0.00	0.01	0.00	0.02	0.02	0.01	0.01	0.01	0.01	0.01
Cerebrovascular disease	0.21	-0.04	0.15	0.06	0.15	1.00	0.13	0.07	0.02	0.12	0.07	0.08	0.25	0.14	0.06	0.04	0.02	0.01	0.01	0.02	0.00	0.01	0.00	0.01	0.01	0.01	0.00	0.00	0.00	0.01
Dementia	0.18	0.02	0.08	0.01	0.03	0.13	1.00	0.03	0.01	0.02	0.01	0.01	0.07	0.01	0.03	0.01	0.01	0.01	0.00	0.02	0.00	0.01	0.00	-0.01	0.01	0.00	0.00	0.01	0.00	0.00
Chronic pulmonary disease	0.10	-0.01	0.10	0.02	0.07	0.07	0.03	1.00	0.05	0.10	0.08	0.03	0.12	0.01	0.04	0.05	0.02	0.02	0.07	0.05	0.01	0.03	0.00	0.06	0.13	0.21	0.03	0.03	0.01	0.03
Rheumatic disease	0.04	0.05	0.04	0.01	0.06	0.02	0.01	0.05	1.00	0.08	0.04	0.01	0.04	0.00	0.02	0.02	0.00	0.00	0.02	0.01	0.01	0.00	0.00	0.01	0.02	0.02	0.01	0.00	0.00	0.01
Peptic ulcer disease	0.11	-0.03	0.12	0.07	0.10	0.12	0.02	0.10	0.08	1.00	0.12	0.05	0.14	0.03	0.05	0.12	0.02	0.01	0.03	0.02	0.01	0.02	0.01	0.02	0.03	0.04	0.01	0.01	0.02	0.01
Liver disease	0.06	-0.05	0.07	0.02	0.07	0.07	0.01	0.08	0.04	0.12	1.00	0.07	0.17	0.01	0.05	0.08	0.01	0.00	0.02	0.02	0.01	0.01	0.01	0.01	0.02	0.02	0.01	0.01	0.01	0.01
Diabetes	0.05	-0.05	0.09	0.06	0.09	0.08	0.01	0.03	0.01	0.05	0.07	1.00	0.11	0.01	0.06	0.04	0.02	0.01	0.01	0.01	0.00	0.01	0.00	0.00	0.00	0.00	0.00	0.00	0.00	0.00
Hypertension	0.27	-0.05	0.22	0.10	0.15	0.25	0.07	0.12	0.04	0.14	0.17	0.11	1.00	0.04	0.11	0.06	0.02	0.01	0.02	0.01	0.00	0.01	0.00	0.00	0.01	0.01	0.00	0.01	0.00	0.01
Hemiplegia or paraplegia	0.02	-0.02	0.02	0.01	0.02	0.14	0.01	0.01	0.00	0.03	0.01	0.01	0.04	1.00	0.01	0.01	0.01	0.00	0.00	0.00	0.00	0.00	0.00	0.00	0.00	0.00	0.00	0.00	0.00	0.00
Renal disease	0.09	-0.05	0.14	0.05	0.06	0.06	0.03	0.04	0.02	0.05	0.05	0.06	0.11	0.01	1.00	0.04	0.01	0.01	0.00	0.01	0.00	0.01	0.00	0.00	0.01	0.00	0.00	0.00	0.00	0.00
Any malignancy^*^	0.11	-0.08	0.06	0.02	0.04	0.04	0.01	0.05	0.02	0.12	0.08	0.04	0.06	0.01	0.04	1.00	0.02	0.02	0.02	0.02	0.00	0.01	0.01	0.01	0.02	0.01	0.01	0.00	0.00	0.00
Penicillins (IV)	0.02	-0.01	0.03	0.01	0.01	0.02	0.01	0.02	0.00	0.02	0.01	0.02	0.02	0.01	0.01	0.02	1.00	0.20	0.03	0.03	0.01	0.03	0.00	0.02	0.04	0.02	0.01	0.02	0.00	0.01
Penicillin BLI (IV)	0.01	-0.01	0.02	0.00	0.01	0.01	0.01	0.02	0.00	0.01	0.00	0.01	0.01	0.00	0.01	0.02	0.20	1.00	0.01	0.03	0.01	0.05	0.00	0.00	0.04	0.01	0.01	0.02	0.01	0.00
Third cephem (oral)	0.01	0.01	0.01	0.01	0.02	0.01	0.00	0.07	0.02	0.03	0.02	0.01	0.02	0.00	0.00	0.02	0.03	0.01	1.00	0.06	0.02	0.03	0.00	0.11	0.08	0.08	0.03	0.01	0.01	0.04
Third cephem (IV)	0.02	-0.01	0.02	0.01	0.01	0.02	0.02	0.05	0.01	0.02	0.02	0.01	0.01	0.00	0.01	0.02	0.03	0.03	0.06	1.00	0.01	0.04	0.01	0.02	0.15	0.06	0.02	0.04	0.01	0.01
Carbapenem (oral)	0.00	0.00	0.00	0.00	0.00	0.00	0.00	0.01	0.01	0.01	0.01	0.00	0.00	0.00	0.00	0.00	0.01	0.01	0.02	0.01	1.00	0.01	0.00	0.02	0.03	0.02	0.01	0.01	0.01	0.01
Carbapenem (IV)	0.02	-0.01	0.01	0.01	0.01	0.01	0.01	0.03	0.00	0.02	0.01	0.01	0.01	0.00	0.01	0.01	0.03	0.05	0.03	0.04	0.01	1.00	0.00	0.02	0.06	0.02	0.04	0.03	0.01	0.02
Aminoglycosides (oral)	0.00	0.00	0.00	0.00	0.00	0.00	0.00	0.00	0.00	0.01	0.01	0.00	0.00	0.00	0.00	0.01	0.00	0.00	0.00	0.01	0.00	0.00	1.00	0.00	0.00	0.00	0.00	0.01	0.00	0.00
Aminoglycosides (IV)	0.01	0.01	0.00	0.00	0.02	0.01	-0.01	0.06	0.01	0.02	0.01	0.00	0.00	0.00	0.00	0.01	0.02	0.00	0.11	0.02	0.02	0.02	0.00	1.00	0.12	0.15	0.03	0.01	0.01	0.03
Quinolones (oral)	0.01	0.03	0.02	0.00	0.02	0.01	0.01	0.13	0.02	0.03	0.02	0.00	0.01	0.00	0.01	0.02	0.04	0.04	0.08	0.15	0.03	0.06	0.00	0.12	1.00	0.12	0.04	0.04	0.02	0.04
Macrolides (oral)	0.00	0.02	0.01	0.00	0.01	0.01	0.00	0.21	0.02	0.04	0.02	0.00	0.01	0.00	0.00	0.01	0.02	0.01	0.08	0.06	0.02	0.02	0.00	0.15	0.12	1.00	0.03	0.03	0.05	0.05
Tetracyclines (oral)	0.00	0.00	0.00	0.00	0.01	0.00	0.00	0.03	0.01	0.01	0.01	0.00	0.00	0.00	0.00	0.01	0.01	0.01	0.03	0.02	0.01	0.04	0.00	0.03	0.04	0.03	1.00	0.08	0.01	0.02
Tetracyclines (IV)	0.01	0.00	0.01	0.00	0.01	0.00	0.01	0.03	0.00	0.01	0.01	0.00	0.01	0.00	0.00	0.00	0.02	0.02	0.01	0.04	0.01	0.03	0.01	0.01	0.04	0.03	0.08	1.00	0.00	0.01
Other (oral)	0.00	0.01	0.00	0.00	0.01	0.00	0.00	0.01	0.00	0.02	0.01	0.00	0.00	0.00	0.00	0.00	0.00	0.01	0.01	0.01	0.01	0.01	0.00	0.01	0.02	0.05	0.01	0.00	1.00	0.04
Other (IV)	0.01	0.01	0.01	0.00	0.01	0.01	0.00	0.03	0.01	0.01	0.01	0.00	0.01	0.00	0.00	0.00	0.01	0.00	0.04	0.01	0.01	0.02	0.00	0.03	0.04	0.05	0.02	0.01	0.04	1.00

In the multivariable Cox regression model (Table 5), adjusted for demographics, comorbid conditions, and baseline antibiotic prescriptions, oral third-generation cephalosporins (HR: 1.14, 95% CI: 1.01-1.29, p = 0.03), tetracyclines (HR: 2.14, 95% CI: 1.47-3.13, p < 0.001), and aminoglycosides (HR: 8.36, 95% CI: 1.18-59.2, p = 0.03) remained statistically significant predictors of ciArAEs among oral antibiotic agents. Furthermore, intravenous penicillins (HR: 1.28, 95% CI: 1.03-1.60, p = 0.02) were associated with a heightened risk of ciArAEs.

**Table 5:** Multivariate Cox regression analysis. IV: intravenous injection.

Variable	Category	Multivariate Cox regression analysis			
		Hazard ratio	95% confidence intervals		p-value
Age	Aged 75 and over	2.56	2.35	2.79	<0.001
Sex	Male	1.56	1.44	1.69	<0.001
Comorbidities					
Congestive heart failure	Presence	1.54	1.37	1.72	<0.001
Myocardial infarction	Presence	0.78	0.58	1.03	0.080
Peripheral vascular disease	Presence	1.37	1.22	1.54	<0.001
Cerebrovascular disease	Presence	1.21	1.10	1.34	<0.001
Dementia	Presence	2.49	2.13	2.91	<0.001
Chronic pulmonary disease	Presence	1.18	1.06	1.31	0.002
Rheumatic disease	Presence	2.01	1.70	2.38	<0.001
Peptic ulcer disease	Presence	1.27	1.14	1.40	<0.001
Liver disease	Presence	1.11	0.99	1.24	0.070
Diabetes	Presence	1.45	1.25	1.69	<0.001
Hypertension	Presence	1.22	1.12	1.33	<0.001
Hemiplegia or paraplegia	Presence	1.90	1.32	2.73	<0.001
Renal disease	Presence	1.55	1.28	1.88	<0.001
Any malignancy	Presence	1.39	1.22	1.58	<0.001
Prescribed antimicrobials					
Penicillins (IV)	Prescribed	1.88	1.11	3.17	0.020
Penicillins with beta-lactamase inhibitors (IV)	Prescribed	0.89	0.32	2.45	0.820
Third-generation cephalosporins (oral)	Prescribed	1.14	1.01	1.29	0.040
Third-generation cephalosporins (IV)	Prescribed	1.44	1.00	2.07	0.050
Carbapenems (oral)	Prescribed	1.66	0.96	2.87	0.070
Carbapenems (IV)	Prescribed	1.49	0.82	2.72	0.190
Aminoglycosides (oral)	Prescribed	8.36	1.18	59.2	0.030
Aminoglycosides (IV)	Prescribed	1.07	0.84	1.35	0.600
Quinolones (oral)	Prescribed	1.09	0.94	1.27	0.240
Macrolides (oral)	Prescribed	1.14	0.99	1.32	0.060
Tetracyclines (oral)	Prescribed	2.14	1.47	3.13	<0.001
Tetracyclines (IV)	Prescribed	1.30	0.53	3.17	0.570
Others (oral)	Prescribed	1.56	0.92	2.65	0.100
Others (IV)	Prescribed	1.48	0.92	2.41	0.110

In the final model, several patient-specific factors emerged as robust predictors of ciArAEs. Older age (HR: 2.56, 95% CI: 2.35-2.79) and male sex (HR: 1.56, 95% CI: 1.44-1.69) were consistently linked to elevated risk. Likewise, comorbidities such as congestive heart failure, peripheral vascular disease, cerebrovascular disease, chronic pulmonary disease, diabetes, and renal disease further increased the likelihood of ciArAEs (all p < 0.05). Taken together, these findings highlight that both particular classes of antibiotics and a patient’s clinical background influence the incidence of antibiotic-related adverse events requiring hospitalization.

## Discussion

In this study, we investigated predictive factors for ciArAEs using Japanese claims data. Our results indicate that third-generation cephalosporins, aminoglycosides, and tetracyclines are potential predictive factors among oral antimicrobials. Furthermore, patient characteristics and existing comorbidities were identified as probable determinants of risk. These findings have significant implications for the risk assessment of antibiotic prescriptions and infectious disease treatments. Healthcare providers, particularly in the context of third-generation cephalosporins, tetracyclines, and aminoglycosides, should consider patient characteristics and existing comorbidities when making decisions. This study offers a novel perspective on drug selection, potentially contributing to the reduction of ciArAEs.

Interestingly, antibiotics such as cephalosporins without oral third-generation cephalosporins, quinolones, and lincomycin, previously suggested to be associated with CDIs [16,17] or infections with drug-resistant bacteria [18], did not augment the risk of ciArAEs. Although prior studies associate macrolides and quinolones with CDI, our study did not find significant associations. This could be due to population differences, coding limitations, or other adjusting factors. This observation may serve as a catalyst to re-evaluate current treatment guidelines based on previous studies.

The effects of age, sex, and comorbidities could be explained by weakened immunity, opportunities for exposure to healthcare institutions, and antimicrobials [19]. Conditions such as diabetes and malignancy have been identified as predictive factors for drug-resistant bacteria [20].

Among antimicrobial agents, intravenous penicillin and orally administered third-generation cephalosporins, tetracyclines, and aminoglycosides were identified as predictive factors. Penicillin, commonly prescribed for diseases with a long duration of treatment such as endocarditis [21] or vertebral osteomyelitis [22], could potentially explain their relatively strong association with ciArAEs. Oral third-generation cephalosporins have low bioavailability, which may promote resistance. Tetracyclines and aminoglycosides have also been linked to bacterial resistance and toxicity. Oral third-generation cephalosporins are a low priority among treatment options for bacterial infections due to their low bioavailability and excessive antimicrobial spectrum. Oral third-generation cephalosporins are considered a low-priority option for treating bacterial infections due to their poor bioavailability and overly broad antimicrobial spectrum [23]. Conversely, these drugs have been linked to drug-resistant bacterial infections and various adverse events [23]; however, few studies have directly examined the risks associated with their prescription. Limited studies suggest tetracyclines are associated with a higher risk of antimicrobial resistance [24], and some indicate a relatively low risk of CDI [25]. However, the association between tetracyclines and CDI is based on small observational studies and their meta-analysis, so the reliability is not robust. Whereas our analysis suggested tetracycline, azithromycin, and other antibiotics as potential contributors to ciArAEs, we emphasize that these findings pertain to a composite outcome. Specific associations with CDI require further focused investigation. CDIs accounted for 23.4% of ciArAEs in this study (Table 2). We caution against drawing immediate conclusions based solely on this broader association and recommend more specific, dedicated studies to substantiate these associations.

One major strength of our study is its innovative approach to differentiating oral and intravenous antimicrobial prescriptions, which has not been thoroughly explored in many prior investigations. Although there are few studies about classes of antibiotics as risk factors for CDI, most of these studies do not distinguish the route of administration [16,17]. Our study serves as a crucial step in filling these gaps. Furthermore, the large-scale nature of the SKDB, covering over 2.6 million individuals across an 8.5-year period, allowed us to capture a wide range of prescribing patterns and patient characteristics. By harnessing such a comprehensive dataset, we were able to explore ciArAE risks more robustly than single-center or smaller cohort studies [6,7].

Overall, our study delves into the intricate relationship between antibiotic usage and associated adverse events, offering a new understanding of antibiotic prescription and utilization. This provides essential information for improving infectious disease treatment strategies and for providing concrete guidelines for the prevention and risk management of ciArAEs. The outcomes of our study not only point the direction for future research into antibiotic use and its associated risks but may also serve as the basis for a re-evaluation of current treatment guidelines.

In light of these findings, the broader context of antibiotic stewardship programs in Japan [26] and worldwide deserves mention. Antibiotic stewardship programs are strategic efforts implemented within healthcare systems to improve and measure the appropriate use of antibiotics. These programs are designed to ensure that patients receive the right antibiotic, dose, time, and duration. The overarching goal of these programs is to improve patient outcomes, reduce microbial resistance, and decrease the spread of infections caused by multidrug-resistant organisms [27]. Understanding the predictive factors associated with ciArAEs, as demonstrated in our study, is vital for the development and success of these programs. For instance, our results indicate that using orally administered third-generation cephalosporins, tetracyclines, and aminoglycosides can be potential predictive factors for ciArAEs, suggesting that these antimicrobials should be used in appropriate situations based on the patient's condition and the assumed pathogenic bacteria. Narrow-spectrum antibiotics may mitigate ciArAE risks. Future studies should explore whether replacing broad-spectrum agents with targeted therapy reduces adverse events. Furthermore, our findings on patient characteristics and comorbidities associated with ciArAEs could be useful in identifying high-risk patient populations and tailoring antibiotic stewardship programs accordingly. Future studies should aim to examine the effectiveness of these stewardship programs in reducing ciArAEs, based on the predictive factors identified in our study.

Limitations

Despite the robust findings of our study, it is crucial to recognize several limitations. As our research relies on ICD-10 coding for identifying ciArAEs, misclassification bias is possible. Some hospitalizations coded as ciArAEs may not have been directly caused by the prescribed antibiotics. No previous studies have validated ciArAEs extraction, as a composite or individual outcome, using these codes. Because these codes may imply high specificity, this study does not overestimate adverse events from widely used antimicrobial agents. Furthermore, our study was constrained by the inherent limitations of the SKDB, leading to the potential exclusion of variables absent from the database, and thus possibly omitting significant risk factors. Excluding individuals without health checkups may have led to selection bias, potentially omitting high-risk patients with frequent hospitalizations or complex medical conditions. Our study does not account for socioeconomic status, healthcare access, or physician prescribing patterns, which may influence both antibiotic use and hospitalization risk. Future studies incorporating these factors are needed. This study establishes an association rather than causation. If the multivariate regression analysis in this study were interpreted as causal inference, the multivariate Cox regression analysis would have adjusted for potential confounding factors, but the potential for residual confounding exists. Future studies using propensity score matching or instrumental variable analysis may help strengthen causal inference.

This study relies on insurance claims data, which lack clinical granularity, such as microbiological test results, laboratory values, and physician-assessed severity scores. Future studies incorporating electronic medical records or microbiological data are needed to validate our findings more comprehensively. While our analysis included a wide spectrum of antibacterial agents as risk factors, it is worth noting that certain recognized risk factors, such as prior hospitalization, were not incorporated due to these limitations. This study did not assess dose-response relationships between antibiotic exposure and ciArAEs. Future research should explore this aspect to better understand risk stratification. The interaction of multiple antimicrobial agents could not be examined. Our study also focused on a specific region of Japan, as dictated by the scope of the SKDB. Therefore, it is important to remember that the incidence of ciArAEs may be impacted by local factors, which calls for careful interpretation when applying our findings to other geographical regions. Lastly, due to the inherent challenges of evaluating an extensive array of drugs utilizing a novel user design, the verification of drug prescription was confirmed within the baseline period. To minimize the bias that could potentially emerge from the inability to account for ciArAEs that occur within a few months post drug administration, we intentionally kept the exploration period for baseline variables to a brief span, specifically three months. However, the results can be influenced by antimicrobial prescriptions during the follow-up period outside the baseline period. Taking this into account may provide a broader understanding of the risks associated with antibiotic-related adverse events. In the future, the history of antibiotic use could be considered a time-dependent variable when assessing the risk of ciArAEs. Despite these limitations, it was clear that our study elucidated the association between the use of specific oral antimicrobial agents and related adverse events.

## Conclusions

In this large, population-based retrospective cohort study using the SKDB, we found that prescriptions for oral third-generation cephalosporins, tetracyclines, and aminoglycosides were associated with a higher risk of ciArAEs requiring hospitalization, particularly among older men and those with multiple comorbidities. These results underscore the need for judicious antibiotic selection and dosing, emphasizing stewardship principles to reduce the emergence of drug resistance and to protect patient health. Addressing these issues through targeted interventions, such as minimizing unnecessary prescriptions, optimizing treatment durations, and enhancing patient counseling, can help mitigate adverse outcomes while bolstering public health measures. Further research aimed at refining prescribing practices, especially in high-risk populations, and periodically revisiting guidelines for appropriate antibiotic use, will be critical to improving patient outcomes and tackling the global threat posed by antimicrobial resistance.

## Appendices

Appendix 1

**Table 6:** ICD-10 codes and disease codes used in this study. * Any malignancy, including lymphoma, leukemia, and malignant neoplasm of the skin. ICD-10: International Classification of Diseases, Tenth Revision.

Covariates	ICD-10 codes
Any malignancy^*^	C00, C01, C02, C03, C04, C05, C06, C07, C08, C09, C10, C11, C12, C13, C14, C15, C16, C17, C18, C19, C20, C21, C22, C23, C24, C25, C26, C30, C31, C32, C33, C34, C37, C38, C39, C40, C41, C43, C45, C46, C47, C48, C49, C50, C51, C52, C53, C54, C55, C56, C57, C58, C60, C61, C62, C63, C64, C65, C66, C67, C68, C69, C70, C71, C72, C73, C74, C75, C76, C81, C82, C83, C84, C85, C88, C90, C91, C92, C93, C94, C95, C96, C97
Myocardial infarction	I21, I22, I252
Cerebrovascular disease	G45, G46, H340, I60, I61, I62, I63, I64, I65, I66, I67, I68, I69
Peripheral vascular disease	I70, I71, I731, I738, I739, I771, I790, I792, K551, K558, K559, Z958, Z959
Hypertension	I10, I11, I12, I13, I15
Liver disease	B18, I85, I864, I982, K70, K711, K713, K714, K715, K717, K72, K73, K74, K760, K762, K763, K764, K765, K766, K767, K768, K769, Z944
Renal disease	I120, I131, N18, N19, N032, N033, N034, N035, N036, N037, N052, N053, N054, N055, N056, N057, N250, Z490, Z491, Z492, Z940, Z992
Diabetes	E100, E101, E109, E110, E111, E119, E120, E121, E129, E130, E131, E139, E140, E141, E149, E102, E103, E104, E105, E107, E112, E113, E114, E115, E117, E122, E123, E124, E125, E127, E132, E133, E134, E135, E137, E142, E143, E144, E145, E147
Rheumatic disease	M05, M06, M08, M120, M123, M30, M310, M311, M312, M313, M315, M32, M33, M34, M35, M360, M45, M461, M468, M469, L940, L941, L943
Congestive heart failure	I099, I110, I130, I132, I255, I420, I425, I426, I427, I428, I429, I43, I50, P290
Dementia	F00, F01, F02, F03, F051, G30, G311
Chronic pulmonary disease	I278, I279, J40, J41, J42, J43, J44, J45, J46, J47, J60, J61, J62, J63, J64, J65, J66, J67, J684, J701, J703
Peptic ulcer disease	K25, K26, K27, K28
Hemiplegia or paraplegia	G041, G114, G801, G802, G81, G82, G830, G831, G832, G833, G834, G839

Appendix 2

**Table 7:** ICD-10 codes and disease codes for ciArAEs. ICD-10: International Classification of Diseases, Tenth Revision; ciArAEs: clinically important antibiotic-related adverse events; BLNAR: β-lactamase negative ampicillin resistant; ESBL: extended-spectrum β-lactamase; MDR: multi-drug resistance; MRCNS: methicillin-resistant coagulase-negative staphylococci; MRSA: methicillin‐resistant *Staphylococcus aureus*; PCG: penicillin-G; PRSP: penicillin-resistant *Streptococcus pneumoniae*; VCM: vancomycin; VRE: vancomycin-resistant enterococci. * Disease code refers to the code used for medical billing in the electronic claims processing system. The corresponding disease name is indicated in parentheses.

Outcome	ICD-10 codes/disease codes*
BLNAR infection	8847067 (BLNAR infection)
Clostridium difficile infection	A047, 5580020 (Pseudomembranous colitis), 8844165 (Pseudomembranous enterocolitis), 8847089 (Clostridium difficile colitis)
ESBL	8847068 (Infection with ESBL-producing bacteria), U822
MDR bacterial infection	8847111 (Multidrug-resistant Acinetobacter infection), 8845583 (Multidrug-resistant enterococcus infection), 8847113 (Multidrug-resistant pseudomonas aeruginosa infection), U837
MRSA infection	8847069 (MRCNS infection), 8847070 (MRCNS pneumonia), 8847071 (MRCNS septicemia), 0381003 (MRSA infection), 8848439 (MRSA shoulder arthritis), 8830115 (MRSA arthritis), 8847072 (MRSA hip Arthritis), 8847832 (MRSA Elbow Arthritis), 8847073 (MRSA knee arthritis), 8830116 (MRSA infective endocarditis), 8830118 (MRSA postoperative wound infection), 8830117 (MRSA osteomyelitis), 8830119 (MRSA meningitis), 8830120 (MRSA colitis), 8830121 (MRSA empyema), 8830122 (MRSA pneumonia), 8830123 (MRSA lung abscess), 8830124 (MRSA septicemia), 8830125 (MRSA peritonitis), 8830126 (MRSA carrier), 8843107 (MRSA cystitis), U821
PCG resistant	U820
PRSP infection	8847138 (PRSP infection)
VCM resistant	U830
VRE infection	8845601 (VRE infection)
